# Genome wide analysis of human genes transcriptionally and post-transcriptionally regulated by the HTLV-I protein p30

**DOI:** 10.1186/1471-2164-10-311

**Published:** 2009-07-14

**Authors:** John M Taylor, Sofiane Ghorbel, Christophe Nicot

**Affiliations:** 1 Center for Viral Oncology, Department of Pathology and Laboratory Medicine, University of Kansas Medical Center, Kansas City, Kansas, 66160, USA

## Abstract

**Background:**

Human T-cell leukemia virus type 1 (HTLV-I) is a human retrovirus that is etiologically linked to adult T-cell leukemia (ATL), an aggressive and fatal lymphoproliferative disease. The viral transactivator, Tax, is thought to play an important role during the initial stages of CD4^+ ^T-cell immortalization by HTLV-1. Tax has been shown to activate transcription through CREB/ATF and NF-KB, and to alter numerous signaling pathways. These pleiotropic effects of Tax modify the expression of a wide array of cellular genes. Another viral protein encoded by HTLV-I, p30, has been shown to affect virus replication at the transcriptional and posttranscriptional levels. Little is currently known regarding the effect of p30 on the expression and nuclear export of cellular host mRNA transcripts. Identification of these RNA may reveal new targets and increase our understanding of HTLV-I pathogenesis. In this study, using primary peripheral blood mononuclear cells, we report a genome wide analysis of human genes transcriptionally and post-transcriptionally regulated by the HTLV-I protein p30.

**Results:**

Using microarray analysis, we analyzed total and cytoplasmic cellular mRNA transcript levels isolated from PBMCs to assess the effect of p30 on cellular RNA transcript expression and their nuclear export. We report p30-dependent transcription resulting in the 2.5 fold up-regulation of 15 genes and the down-regulation of 65 human genes. We further tested nuclear export of cellular mRNA and found that p30 expression also resulted in a 2.5 fold post-transcriptional down-regulation of 90 genes and the up-regulation of 33 genes.

**Conclusion:**

Overall, our study describes that expression of the HTLV-I protein p30 both positively and negatively alters the expression of cellular transcripts. Our study identifies for the first time the cellular genes for which nuclear export is affected by p30. These results suggest that p30 may possess a more global function with respect to mRNA transcription and the nuclear shuttling of cellular mRNA transcripts. In addition, these alterations in gene expression may play a role in cell transformation and the onset of leukemia.

## Background

Human T-cell leukemia virus type 1 (HTLV-I) was identified in 1980 as the first oncogenic human retrovirus. Infection is associated with adult T-cell leukemia (ATL) as well as other pathological disorders such as tropical spastic paraparesis/HTLV-1 associated myelopathy (TSP/HAM) [1-3]. It is estimated that 25 million people worldwide are infected with HTLV-I. HTLV-I is endemic in Japan, the Caribbean, South America and Central Africa. The majority of carriers will remain asymptomatic throughout their entire lives, and data from Japan has estimated that approximately 6.6% of infected males and 2.1% of infected females will ultimately develop ATL during their lifespan [4]. HTLV-I is transmitted vertically from mother to child prior to birth or through contaminated blood products. Intriguingly, individuals infected with HTLV-1 later in life rarely present with ATL, suggesting that infection during infancy is an important factor for the development of ATL.

HTLV-I is a relatively small virus with a genome only 9 kb in size that encodes for the classic retroviral structural proteins gag, pol, and env. Downstream of the env gene lies a unique region, named pX, which encodes for a number of non-structural proteins involved in virus gene expression and cell transformation, including Tax, Rex, p30, p12, p13, and HBZ. Replication of HTLV-I is dependent on the expression of the viral transactivating protein, Tax, which recruits CREB, CBP/p300 and PCAF, to activate viral gene transcription from the viral LTR [5-11]. As Tax has been implicated in the activation of a number of cell survival pathways, it is believed to be primarily responsible for facilitating cell transformation and the onset of leukemia. Tax, however, is highly immunogenic and cells expressing high amounts of Tax are readily detected and cleared by the human immune system. As a result, cells that express low amounts of Tax are preferentially selected in vivo; in fact, ATL cells from leukemic patients do not express detectable viral proteins. This selective mechanism is apparent as patients who develop ATL exhibit a clonal pattern of provirus integration and T-cell receptor rearrangement, indicating that ATL arises from a single infected progenitor cell.

Other HTLV-I non-structural proteins appear to regulate various steps of virus gene expression, often through unique mechanisms. Rex is an RNA binding protein that binds to an RNA responsive element located at the 3' terminus of all viral mRNA molecules to facilitate RNA export to the cytoplasm [12-14]. In contrast to Tax and Rex which positively regulate virus gene expression, two other viral proteins have been shown to negatively regulate gene expression. HBZ, the HTLV basic leucine zipper protein, is an anti-sense-encoded protein that interferes with Tax-mediated viral gene expression [15,16]. Another intriguing viral protein, p30, is a post-transcriptional repressor of gene expression [17,18]. Specifically, p30 expression inhibits the export of the tax/rex mRNA message from the nucleus [17]. Unlike Rex, p30 does not shuttle between the nucleus and the cytoplasm and is predominantly localized to the nucleus/nucleolus, consistent with a nuclear RNA retention mechanism and function [19]. p30 interacts with Rex, and this interaction is enhanced in the presence of viral RNA, suggesting that p30 may function by binding Rex in complex with viral RNA, thereby inhibiting Rex-dependent RNA export [20]. It is not known, however, whether this interaction is required for the ability of p30 to inhibit Tax mRNA export from the nucleus.

Recently, it has been shown that p30 expression also influences the cell cycle and TLR signalling [21,22]. A separate gene profile experiment suggested that p30 was able to specifically down-regulate a variety of cellular genes involved in apoptosis, cell cycle, and transcription [23]. Whether these effects are required for HTLV-I infectivity or cell transformation remains to be seen, although studies have shown that p30 expression is required for efficient virus replication and persistence in vivo [24,25].

While some information is known regarding the effects of p30 on gene transcription, the effects of p30 on cellular mRNA nuclear export have not been investigated. Using differential gene microarray analysis on total and cytoplasmic RNA extracts from peripheral blood mononuclear cells expressing p30 in comparison with mock-transduced cells, we assessed the relative abundance of cellular transcripts regulated at both the transcriptional and post transcriptional level. We found that p30 alters the expression and nucleo-cytoplasmic localization of a number of cellular transcripts involved in RNA processing, cell signalling, metabolism, and cell division. These results will help pave the way for future studies aimed at understanding the role of these cellular RNA in HTLV-I pathogenesis.

## Results

### p30 transcriptionally regulates the expression level of various cellular mRNAs

**Figure 1:**
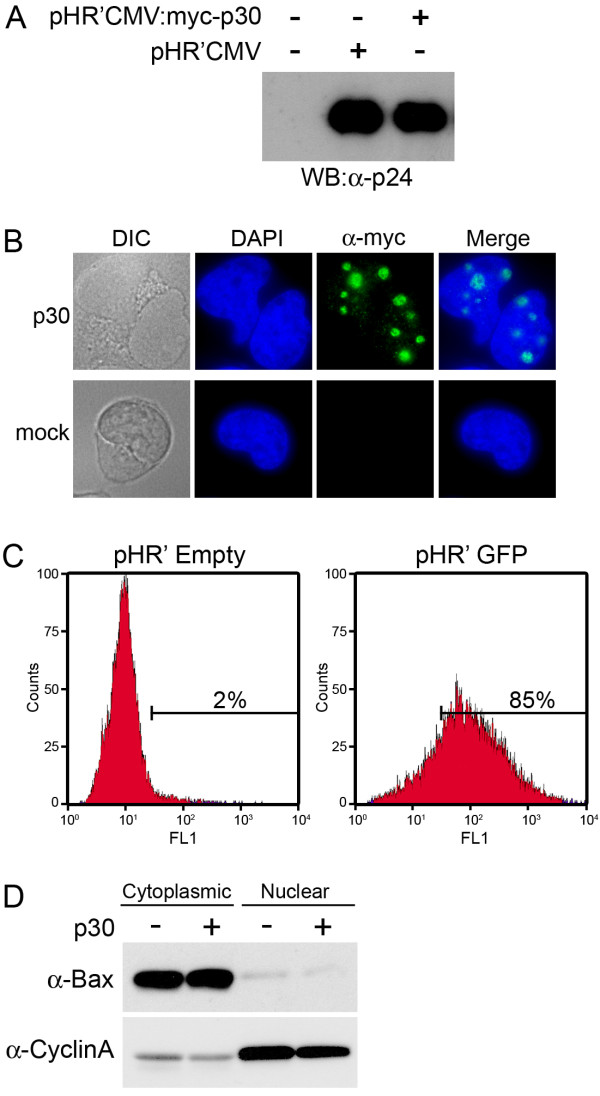
**Production of infectious lentiviral particles from transfected 293 T cells**. A) 293 T cells were mock transfected, or transfected with pDNL6, pVSV-G, and either pHR'CMV or pHR'CMV:myc-p30, and supernatants were western blotted for HIV p24 Gag expression. B) Lymphocytes co-cultured with 293 T producer cells transfected with either pHR'CMV or pHR'CMV:myc-p30 were harvested, fixed and stained with anti-myc (9E10) and anti-mouse-Alexa488, and visualized by immunofluorescence. C) Lymphocytes co-cultured with 293 T cells transfected with either pHR'CMV or pHR'CMV:GFP were fixed and analyzed for GFP expression by flow cytometry. D) Cytosolic or nuclear protein fractions were isolated following cytoplasmic RNA extraction, and were analyzed by western blotting with either anti-cyclin A or anti-Bax to assess nuclear and cytoplasmic fraction purity, respectively.

To analyze the transcriptional effects of p30 on global gene expression, we isolated RNA from total RNA pools following p30 expression in PBMCs using a lentiviral system. Cells were co-transfected with a packaging vector pDNL6, VSV-G to pseudotype lentiviral particles, and either empty vector pHR'CMV, or pHR'CMV:myc-p30. To ensure equivalent virus production from cells transfected with either pHR'CMV or pHR'CMV:myc-p30, we assessed the amount of lentiviral particles released into the supernatant by analyzing supernatants for HIV p24 Gag, which is a structural protein packaged as part of the lentiviral particle. Cell supernatants following transfection were harvested, lysed in SDS sample buffer and separated by SDS-PAGE. Western blotting with anti-p24 showed an equivalent amount of p24 in the supernatants of cells transfected with either the control plasmid pHR'CMV or pHR'CMV:myc-p30 (Figure 1A). We also assessed the expression of p30 by staining lentivirus-infected cells with anti-myc to detect p30 by immunofluorescence. As expected, nucleolar p30 was observed, indicating that p30 was expressed following infection of co-cultured cells (Figure 1B). To determine the percentage of cells infected using our lentiviral packaging and infection system, we co-transfected cells with pHR'CMV:GFP, which expresses GFP. Following lentiviral infection, greater than 85% of cells expressed GFP, indicating that our lentiviral packaging system is highly effective (Figure 1C).

To examine RNA transcript levels in PBMCs in the presence and absence of p30, lentivirus-producing 293 T cells were co-cultured with peripheral blood mononuclear cells for 48 hours, total RNA was isolated and RNA samples were subjected to microarray analysis. Following microarray analysis, data sets were analyzed using the following steps: transcripts significantly present in the total RNA fraction from cells transduced with pHR'CMV lacking p30 were first selected. This data set was then sorted based on a decrease in transcript abundance following expression of p30. Sixty-five probes exhibited a decrease of greater than 2.5 fold and were deemed to be down-regulated in the presence of p30 (Table 1), and an additional 678 were down-regulated more than 1.5 fold (see Additional file 1). These genes were found to belong to several categories, such as cell signalling, transcription/translation, cell cycle, and metabolism.

**Table 1:** Genes down-regulated in the total RNA fraction in the presence of HTLV-I p30

**Probe ID**	**Gene Description**	**Gene**	**Fold Change**
	**Transcription/Translation/RNA Processing**		
1554468_s_at	Mitochondrial Ribosomal Protein L38	FBF1	0.11
217340_at	Ribosomal Protein L21	RPL21	0.16
231658_x_at	Similar to Ribosomal Protein L36	LOC127295	0.19
243560_at	Heat Shock Transcription Factor 1	HSF1	0.24
216282_x_at	Polymerase (RNA) II (DNA Directed) Polypeptide C, 33 kda	POLR2C	0.25
236522_at	Nuclear Factor I/A	NFIA	0.27
1553181_at	DEAD (Asp-Glu-Ala-Asp) Box Polypeptide 31	DDX31	0.27
216117_at	Exosome Component 2	EXOSC2	0.32
208345_s_at	Pou Domain, Class 3, Transcription Factor 1	POU3F1	0.37
1552499_a_at	Zinc Finger Protein 31 (Kox 29)	ZSCAN20	0.38
	**Metabolism**		
1569631_at	Nicotinamide Nucleotide Adenylyltransferase 1	NMNAT1	0.05
217203_at	Glutamate-Ammonia Ligase (Glutamine Synthetase)	GLUL	0.09
226209_at	NADH Dehydrogenase (Ubiquinone) Flavoprotein 3, 10 kda	NDUFV3	0.16
239333_x_at	Glutathione S-Transferase Subunit 13 Homolog	GSTK1	0.31
226947_at	Glucuronidase, Beta-Like 2	GUSBL2	0.34
213935_at	Abhydrolase Domain Containing 5	ABHD5	0.35
238724_at	2,3-Bisphosphoglycerate Mutase	BPGM	0.37
213582_at	ATPase, Class VI, Type 11a	ATP11A	0.38
236018_at	Adenosine Deaminase-Like	ADAL	0.40
	**Signal Transduction**		
1555340_x_at	Rap1a, Member Of Ras Oncogene Family	RAP1A	0.00
218711_s_at	Serum Deprivation Response (Phosphatidylserine Binding Protein)	SDPR	0.05
210374_x_at	Prostaglandin E Receptor 3 (Subtype Ep3)	PTGER3	0.12
216979_at	Nuclear Receptor Subfamily 4, Group A, Member 3	NR4A3	0.13
242875_at	Presenilin 1 (Alzheimer Disease 3)	PSEN1	0.13
204936_at	Mitogen-Activated Protein Kinase Kinase Kinase Kinase 2	MAP4K2	0.25
211471_s_at	Rab36, Member Ras Oncogene Family	RAB36	0.28
215134_at	Phosphatidylinositol 4-Kinase Type Ii	PI4K2A	0.33
219738_s_at	Protocadherin 9	PCDH9	0.35
239202_at	Rab3b, Member Ras Oncogene Family	RAB3B	0.36
205280_at	Glycine Receptor, Beta	GLRB	0.37
209101_at	Connective Tissue Growth Factor	CTGF	0.37
	**Cell Division/Cell Cycle**		
227578_at	Thymopoietin	TMPO	0.15
219831_at	Cyclin-Dependent Kinase-Like 3	CDKL3	0.29
	**Cytoskeleton**		
239067_s_at	Pannexin 2	PANX2	0.16
206117_at	Tropomyosin 1 (Alpha)	TPM1	0.31
205328_at	Claudin 10	CLDN10	0.33
	**Transport**		
216504_s_at	Solute Carrier Family 39 (Zinc Transporter), Member 8	SLC39A8	0.11
242554_at	Two Pore Segment Channel 2	TPCN2	0.40
	**Apoptosis**		
202730_s_at	Programmed Cell Death 4 (Neoplastic Transformation Inhibitor)	PDCD4	0.34
211603_s_at	Ets Variant Gene 4 (E1A Enhancer Binding Protein, E1af)	ETV4	0.34
244035_at	B-Cell Cll/Lymphoma 2	BCL2	0.36
	**Ubiquitin**		
241900_at	Smad Specific E3 Ubiquitin Protein Ligase 2	SMURF2	0.20
243216_x_at	Ubiquitin Specific Peptidase 40	USP40	0.38
	**Other/Unknown**		
233351_at	DNAI (Hsp40) Homolog, Subfamily C, Member 3	DNAJC3	0.08
243221_at	Family With Sequence Similarity 20, Member A	FAM20A	0.08
236378_at	Calcium And Integrin Binding Family Member 4	CIB4	0.09
222109_at	Guanine Nucleotide Binding Protein-Like 3 (Nucleolar)-Like	GNL3L	0.09
243989_at	Ku-Mel-3 Protein	KU-MEL-3	0.13
221225_at	Dephospho-Coa Kinase Domain Containing	DCAKD	0.13
229678_at	Male-Enhanced Antigen 1	MEA1	0.15
1552430_at	WD Repeat Domain 17	WDR17	0.24
211113_s_at	ATP-Binding Cassette, Sub-Family G (White), Member 1	ABCG1	0.28
207408_at	Solute Carrier Family 22 (Organic Cation Transporter), Member 14	SLC22A14	0.30
233532_x_at	Intraflagellar Transport 52 Homolog (Chlamydomonas)	IFT52	0.34
214181_x_at	Leukocyte-Specific Transcript 1	LST1	0.35
203404_at	Armadillo Repeat Containing, X-Linked 2	ARMCX2	0.36
203765_at	Grancalcin, Ef-Hand Calcium Binding Protein	GCA	0.36
1555046_at	FSH Primary Response (Lrpr1 Homolog, Rat) 1	CENPI	0.37
235991_at	CUB Domain Containing Protein 2	CDCP2	0.37
1554112_a_at	Unc-51-Like Kinase 2 (C. Elegans)	ULK2	0.37
235349_at	Family With Sequence Similarity 82, Member A	FAM82A	0.38
206928_at	Zinc Finger Protein 124 (Hzf-16)	ZNF124	0.39
236943_at	Lymphocyte Antigen 86	LY86	0.39
242996_at	Mitochondrial Translational Release Factor 1	MTRF1	0.40

Among the genes down-regulated by p30 was the gene encoding anti-apoptotic Bcl-2, which is noteworthy since HTLV-1-infected cells are highly resistant to apoptosis. Bcl-2 levels, however, are not known to be elevated in HTLV-infected cells, as other anti-apoptotic proteins appear to be involved in protecting cells from apoptosis [26-28]. Another apoptosis-related gene that was down-regulated, programmed cell death-4 (PDCD4) [29], inhibits protein synthesis by suppression of translation initiation by targeting eIF-4A. In addition, loss of PDCD4 expression in human lung cancer cells has been shown to correlate with tumor progression and poor prognosis[30]. We also found that p30 down-regulates expression of Presenilin 1, a critical component of the gamma-secretase complex, which has been documented to be involved in Notch signalling [31]. Decreased expression of Presenilin 1 is associated with resistance of acute T-cell lymphoblastic leukemia (ALL) to gamma-secretase inhibitors (GSI). We have previously reported that p30 nucleolar retention is in part due to its association with the 60S ribosomal large subunit component L23A. The present study also reveals that expression of several ribosomal proteins is reduced upon p30 expression, including mitochondrial ribosomal protein L38, similar to ribosomal protein L36, and ribosomal protein L21. Rab guanine nucleotide exchange factor (GEF) and the binding partner of phosphatidyl inositol 4-kinase (PI4K) were both down-regulated by p30. Although there is no information known on HTLV-I and PI4K, HTLV infected cells have constitutive activation of PI3K [32,33]. We also observed a decrease in the expression of a number of zinc finger proteins, which are also commonly down-regulated in ATL samples [34].

We next assessed whether p30 expression resulted in the transcriptional up-regulation of any cellular transcripts. Data sets from the above samples were instead selected based on the presence of a particular transcript in the total RNA of p30-expressing cells. This data was then sorted based on an increase in transcript abundance following p30 expression as compared to samples transduced with the pHR'CMV lentivirus alone. Those probes exhibiting greater than a 2.5 fold change in abundance in the presence of p30 were deemed to be up-regulated. Interestingly, only 15 genes were up-regulated, with the largest group of genes being those involved in transcription/translation and RNA processing (Table 2). One of these genes, FOXC2, has been implicated in angiogenesis and cell migration [35]. While very few genes were seen to be up-regulated more than 2.5 fold, close to 500 genes were up-regulated between 1.5 and 2.5 fold (see Additional file 2). Interestingly, several of these genes identified are also known to be up-regulated in acute ATL, including a 90 kDa heat-shock protein, RNA polymerase II (DNA directed), regulator of G-protein signalling, [34], and general transcription factor IIH [36]. Of note, RAG-1 is up-regulated in our p30 extracts, but is conversely seen down-regulated in Tax microarray analysis [37], while Bcl-3, up-regulated in our screen (Table 2), is also up-regulated by Tax [37].

**Table 2:** Genes up-regulated in the total RNA fraction in the presence of HTLV-I p30

**Probe ID**	**Gene Description**	**Gene**	**Fold Change**
	**Transcription/Translation/RNA Processing**		
224442_AT	PHD Finger Protein 6	PHF6	3.2
219730_AT	Mediator Of RNA Polymerase II Transcription, Subunit 18 Homolog (Yeast)	MED18	2.7
1560981_A_AT	Peroxisome Proliferative Activated Receptor, Alpha	PPARA	2.6
239058_AT	Forkhead Box C2 (Mfh-1, Mesenchyme Forkhead 1)	FOXC2	2.6
	**Metabolism**		
210050_AT	Triosephosphate Isomerase 1	TPI1	3.6
224042_AT	Ureidopropionase, Beta	UPB1	2.6
	**Transport**		
243166_AT	Solute Carrier Family 30 (Zinc Transporter), Member 5	SLC30A5	3.0
	**Cell Adhesion**		
227209_AT	Contactin 1	CNTN1	2.5
	**Ubiquitin/Protein Degradation**		
1569706_AT	Myb-Like, Swirm And Mpn Domains 1	MYSM1	2.9
203758_AT	Cathepsin O	CTSO	2.6
	**Other/Unknown**		
228493_AT	A Kinase (Prka) Anchor Protein 14	AKAP14	4.3
1559048_AT	Bah Domain And Coiled-Coil Containing 1	BAHCC1	2.6
226489_AT	Transmembrane And Coiled-Coil Domain Family 3	TMCC3	2.6
1554237_AT	Serologically Defined Colon Cancer Antigen 8	SDCCAG8	2.5
227546_X_AT	Aurora Kinase A Interacting Protein 1	AURKAIP1	2.5

### p30 post-transcriptionally regulates the levels of certain cellular mRNA transcripts

It has been demonstrated that p30 specifically inhibits the export of tax/rex mRNA molecules from the nucleus by an unknown mechanism [17]. Although tax/rex mRNA is the only known transcript which is retained in the nucleus by p30, we hypothesized that other cellular transcripts might also be subject to this virus-mediated RNA retention mechanism. To investigate whether the export of any cellular RNA transcripts from the nucleus to the cytoplasm was inhibited by p30, we isolated RNA from the cytoplasmic fraction of cells in parallel with our total RNA extractions described above. To ascertain the relative purity of cytoplasmic fractions, proteins were isolated following RNA extraction and analyzed by western blotting for known nuclear and cytoplasmic proteins, Cyclin A and Bax, respectively [38,39]. The cytoplasmic fraction was relatively free of cyclin A, while the nuclear fraction was correspondingly free of cytoplasmic Bax protein, as expected (Figure 1D).

Extracted RNA samples were then subjected to microarray analysis. To identify those transcripts that p30 might inhibit the export of, we first selected transcripts that were present in equal abundance in total RNA fractions from cells either plus or minus p30. The data were then re-sorted based on the relative decrease in abundance of transcripts in the cytoplasmic fraction in the presence of p30. By ensuring that transcripts were present in the total RNA fraction in both the presence and absence of p30, any difference seen in cytoplasmic abundance of a particular transcript would not be the result of a general inhibition of transcription by p30. This parallels the repression of Tax mRNA export, where Tax mRNA is decreased in cytoplasmic RNA fractions in the presence of p30, but total Tax mRNA levels are unchanged [17]. In our screen, 90 transcripts were decreased by more than 2.5 fold (Table 3), and another 650 transcripts showed a decreased cytoplasmic abundance of more than 1.5 fold in the presence of p30 (see Additional file 3). Classes of transcripts exhibiting lower abundance in the cytoplasm in the presence of p30 include genes involved in transcription/translation, cell signalling, the cytoskeleton, DNA repair and replication, and metabolism.

**Table 3:** Genes down-regulated in the cytoplasm in the presence of HTLV-I p30

**Probe ID**	**Gene Description**	**Gene**	**Fold Change**
	**Transcription/Translation/RNA Processing**		
209060_X_AT	Nuclear Receptor Coactivator 3	NCOA3	0.11
242113_AT	A Kinase (Prka) Anchor Protein 8-Like	AKAP8L	0.11
228711_AT	Zinc Finger Protein 37a (Kox 21)	ZNF37A	0.14
238185_AT	RNA Binding Motif, Single Stranded Interacting Protein 1	RBMS1	0.20
223409_AT	Forkhead Box K2	FOXK2	0.23
1557813_AT	Single-Stranded DNA Binding Protein 2	SSBP2	0.29
213837_AT	Lethal (3) Malignant Brain Tumor L(3)Mbt Protein (Drosophila) Homolog	L3MBTL	0.31
240482_AT	Histone Deacetylase 3	HDAC3	0.32
203674_AT	Helicase With Zinc Finger	HELZ	0.37
242407_AT	Arginine-Glutamic Acid Dipeptide (Re) Repeats	RERE	0.38
212720_AT	Poly(A) Polymerase Alpha	PAPOLA	0.38
1562741_AT	Ubx Domain Containing 2	UBXD2	0.39
213756_S_AT	Heat Shock Transcription Factor 1	HSF1	0.40
	**Metabolism**		
213935_AT	Abhydrolase Domain Containing 5	ABHD5	0.04
237849_AT	Mannosidase, Alpha, Class 1a, Member 1	MAN1A1	0.17
208917_X_AT	NAD Kinase	NADK	0.24
1570165_AT	Carbohydrate (Chondroitin 4) Sulfotransferase 11	CHST11	0.33
238114_AT	Protein-L-Isoaspartate (D-Aspartate) O-Methyltransferase Domain Containing 1	PCMTD1	0.34
236742_AT	ADP-Ribosylation Factor Guanine Nucleotide-Exchange Factor 1(Brefeldin A-Inhibited)	ARFGEF1	0.35
210963_S_AT	Glycogenin 2	GYG2	0.36
223142_S_AT	Uridine-Cytidine Kinase 1	UCK1	0.37
243501_AT	ATP Synthase, H+ Transporting, Mitochondrial F0 Complex, Subunit B1	ATP5F1	0.37
207904_S_AT	Leucyl/Cystinyl Aminopeptidase	LNPEP	0.38
203180_AT	Aldehyde Dehydrogenase 1 Family, Member A3	ALDH1A3	0.40
	**Cell Signaling/Immune System**		
234628_AT	Rab28, Member Ras Oncogene Family	RAB28	0.11
201295_S_AT	WD Repeat And Socs Box-Containing 1	WSB1	0.18
235213_AT	Inositol 1,4,5-Trisphosphate 3-Kinase B	ITPKB	0.19
229895_S_AT	Nck Adaptor Protein 1	NCK1	0.25
223674_S_AT	CDC42 Small Effector 1	CDC42SE1	0.28
205988_AT	CD84 Antigen (Leukocyte Antigen)	CD84	0.28
242946_AT	CD53 Antigen	CD53	0.30
226375_AT	Lemur Tyrosine Kinase 2	LMTK2	0.32
203854_AT	Complement Factor I	CFI	0.36
1569022_A_AT	Phosphoinositide-3-Kinase, Class 2, Alpha Polypeptide	PIK3C2A	0.37
204813_AT	Mitogen-Activated Protein Kinase 10	MAPK10	0.38
235419_AT	Erbb Receptor Feedback Inhibitor 1	ERRFI1	0.39
	**Cell Division/DNA Replication & Repair**		
229548_AT	Unc-84 Homolog B (C. Elegans)	UNC84B	0.07
1557830_AT	Cyclin J	CCNJ	0.28
	**Cytoskeleton**		
211089_S_AT	Nima (Never In Mitosis Gene A)-Related Kinase 3	NEK3	0.13
243988_AT	Tubulin Tyrosine Ligase-Like Family, Member 5	TTLL5	0.14
240363_AT	Ankyrin 1, Erythrocytic	ANK1	0.16
217297_S_AT	Myosin IXb	MYO9B	0.18
1565149_AT	Dynein, Cytoplasmic 2, Heavy Chain 1	DYNC2H1	0.31
236437_AT	Laminin, Beta 1	LAMB1	0.34
239170_AT	Arp3 Actin-Related Protein 3 Homolog (Yeast)	ACTR3	0.37
215910_S_AT	Fibronectin Type III Domain Containing 3a	FNDC3A	0.38
	**Transport**		
1553148_A_AT	Sorting Nexin 13	SNX13	0.12
233420_AT	Nucleoporin 133 kda	NUP133	0.27
207594_S_AT	Synaptojanin 1	SYNJ1	0.30
203106_S_AT	Vacuolar Protein Sorting 41 (Yeast)	VPS41	0.31
212921_AT	Set And Mynd Domain Containing 2	SMYD2	0.35
1559862_AT	Coatomer Protein Complex, Subunit Alpha	COPA	0.37
244219_AT	Wilms Tumor 1 Associated Protein	WTAP	0.39
	**Apoptosis**		
1562111_AT	Brain And Reproductive Organ-Expressed (Tnfrsf1a Modulator)	BRE	0.01
241876_AT	MDM4, Transformed 3T3 Cell Double Minute 4, p53 Binding Protein	MDM4	0.27
	**Other/Unknown**		
227425_AT	Ralbp1 Associated Eps Domain Containing 2	REPS2	0.06
244571_S_AT	Tetratricopeptide Repeat Domain 12	TTC12	0.08
242870_AT	Family With Sequence Similarity 80, Member B	FAM80B	0.10
222566_AT	Suppressor Of Variegation 4–20 Homolog 1 (Drosophila)	SUV420H1	0.10
240149_AT	Amplified In Breast Cancer 1	HEATR6	0.11
232304_AT	Pellino Homolog 1 (Drosophila)	PELI1	0.13
238995_AT	Sulfotransferase Family, Cytosolic, 1a, Phenol-Preferring, Member 1	SULT1A1	0.18
231437_AT	Solute Carrier Family 35, Member D2	SLC35D2	0.19
242662_AT	Proprotein Convertase Subtilisin/Kexin Type 6	PCSK6	0.20
1557394_AT	Discs, Large (Drosophila) Homolog-Associated Protein 4	DLGAP4	0.20
238011_AT	Pro0149 Protein	C16ORF72	0.20
241268_X_AT	SAM Domain And HD Domain 1	SAMHD1	0.21
222038_S_AT	WD Repeat Domain 50	UTP18	0.22
231003_AT	Solute Carrier Family 35, Member B3	SLC35B3	0.22
242268_AT	Cug Triplet Repeat, RNA Binding Protein 2	CUGBP2	0.26
202516_S_AT	Discs, Large Homolog 1 (Drosophila)	DLG1	0.26
1556839_S_AT	Spectrin, Beta, Non-Erythrocytic 5	SPTBN5	0.27
221430_S_AT	Ring Finger Protein 146	RNF146	0.28
1558941_AT	Zinc Finger Protein 704	ZNF704	0.29
211392_S_AT	Zinc Finger Protein 278	PATZ1	0.29
216604_S_AT	Solute Carrier Family 7 (Cationic Amino Acid Transporter, Y+ System), Member 8	SLC7A8	0.29
240125_AT	Dystrobrevin, Alpha	DTNA	0.30
207781_S_AT	Zinc Finger Protein 6 (Cmpx1)	ZNF711	0.30
1559078_AT	B-Cell Cll/Lymphoma 11a (Zinc Finger Protein)	BCL11A	0.31
237747_AT	Family With Sequence Similarity 107, Member B	FAM107B	0.32
1570165_AT	Carbohydrate (Chondroitin 4) Sulfotransferase 11	CHST11	0.33
219846_AT	Gon-4-Like (C.Elegans)	GON4L	0.36
209766_AT	Peroxiredoxin 3	PRDX3	0.37
1556538_AT	Antigen P97 (Melanoma Associated) Identified By Monoclonal Antibodies 133.2 And 96.5	MFI2	0.37
243367_AT	Dopamine Receptor D5 Pseudogene 2	DRD5P2	0.37
1552678_A_AT	Ubiquitin Specific Peptidase 28	USP28	0.38
236603_AT	WD Repeat Domain 32	WDR32	0.38
224703_AT	WD Repeat Domain 22	WDR22	0.39
232362_AT	Coiled-Coil Domain Containing 18	CCDC18	0.40
213748_AT	Tripartite Motif-Containing 66	TRIM66	0.40

Of the genes we identified, those of particular interest with respect to HTLV and the onset of leukemia include MDM4, a known regulator of p53 [40], and the single-stranded nucleic acid binding protein RBMS1, which has also been linked to transcription and apoptosis [41]. We also found that histone deacetylase HDAC3 was repressed by p30. HDAC3 is known to form multi-protein complexes with the co-repressors SMRT and N-CoR and regulates the transcription of numerous genes [42,43]. In addition, HDAC3 has multiple functions that relate to HTLV-I infected cells. HDAC3 regulates the duration of NF-kB activation by deacetylation of RelA thereby promoting its interaction with inhibitory-kB (IkB) and termination of NF-kB signalling [44]. HDAC3 has also been found to deacetylate acetyltransferases PCAF and p300/CBP and inhibit their function [45,46]. Since both PCAF and p300/CBP are required for Tax-mediated viral transcription, it makes sense that p30 would block this pathway. Finally, downregulation of HDAC3 below threshold induces G2/M arrest, a phenotype previously observed in cells overexpressing p30 [21,47]. These observations warrant further studies. Our results also indicate a decreased expression of Nup133, a nucleoporin protein involved in mRNA export [48]. This is of particular interest because of our previous work demonstrating a nuclear retention of tax/rex mRNA by p30 that is currently under investigation [17].

We then re-sorted the data to examine whether any genes showed an increased abundance in the cytoplasm in the presence of p30. Intriguingly, despite the role of p30 as an inhibitor of Tax mRNA export, we observed 33 genes that showed an increased abundance of more than 2.5 fold in the cytoplasm in the presence of p30 (Table 4), and another 930 genes increased in the cytoplasm more than 1.5 fold (see Additional file 4). These probes were present in equal quantities in the total RNA fractions, indicating that their increased abundance in the cytoplasm was not due to an altered expression pattern at the transcriptional level. Of particular interest are groups of genes involved in DNA replication and repair, apoptosis, cell adhesion, and cell signalling, all of which are known classes of genes to be intimately linked with the onset of cancer. Again, none of the genes identified in our screen were previously shown to be significantly altered in ATL cells [34]. Upregulated genes include two Rab GTPases, which are believed to play a role in oncogenesis [49], and CCAR1, which is involved in gene expression and functions as a p53 coactivator [50]. Other genes observed to be increased between 1.5 and 2.5 fold include XRCC1 binding protein-1, which parallels a previous observation describing an increase in abundance of XRCC1 DNA repair gene in ATL cells [36]. We also observed granzyme B and perforin to be up-regulated in the cytoplasm of p30-expressing cells, contrasting a decrease in the expression of granzyme A in ATL cells [34]. Whether these cytotoxic molecules are involved in the pathogenesis or development of disease in HTLV-I-infected T-cells remains to be investigated.

**Table 4:** Genes up-regulated in the cytoplasm in the presence of HTLV-I p30

**Probe ID**	**Gene Description**	**Gene**	**Fold Change**
	**Transcription/Translation**		
236566_AT	Cell Division Cycle And Apoptosis Regulator 1	CCAR1	2.8
230998_AT	Chromobox Homolog 3 (Hp1 Gamma Homolog, Drosophila)	CBX3	2.7
236244_AT	Heterogeneous Nuclear Ribonucleoprotein U (Scaffold Attachment Factor A)	HNRNPU	2.6
	**Metabolism**		
233445_AT	Bub1 Budding uninhibited by Benzimidazoles 1 Homolog (Yeast)	BUB1	2.5
221139_S_AT	Cysteine Sulfinic Acid Decarboxylase	CSAD	2.7
204646_AT	Dihydropyrimidine Dehydrogenase	DPYD	3.2
242235_X_AT	Nardilysin (N-Arginine Dibasic Convertase)	NRD1	2.6
229465_S_AT	Protein Tyrosine Phosphatase, Receptor Type, D	PTPRS	2.7
242905_AT	Putatative 28 KDa Protein	PNO1	3.1
38964_R_AT	Wiskott-Aldrich Syndrome (Eczema-Thrombocytopenia)	WAS	2.6
	**Signal Transduction**		
1552312_A_AT	Microfibrillar-Associated Protein 3	MFAP3	2.5
220500_S_AT	Rab, Member of Ras Oncogene Family-Like 2a	RABL2A	4.1
220500_S_AT	Rab, Member of Ras Oncogene Family-Like 2b	RABL2B	4.1
239031_AT	Somatostatin Receptor 2	SSTR2	2.9
	**Cell Division/DNA Replication**		
234605_AT	CDC14 Cell Division Cycle 14 Homolog B (S. Cerevisiae)	CDC14B	3.0
1566043_AT	Inner Centromere Protein Antigens 135/155 kda	INCENP	2.9
228670_AT	Telomerase-Associated Protein 1	TEP1	2.5
	**Transport**		
1558028_X_AT	Translocation Associated Membrane Protein 1	TRAM1	2.7
	**Development**		
224368_S_AT	Ndrg Family Member 3	NDRG3	2.5
217107_AT	Ribosomal Protein S4, X-Linked	RPS4X	2.6
232975_AT	Tripartite Motif-Containing 26	TRIM26	2.5
	**Apoptosis**		
40489_AT	Atrophin 1	ATN1	2.9
	**Other/Unknown**		
219301_S_AT	Contactin Associated Protein-Like 2	CNTNAP2	2.8
230701_X_AT	Kinesin Family Member 9	KIF9	2.9
241385_AT	Dkfzp564k112 Protein	LARP7	2.7
226078_AT	RNA Pseudouridylate Synthase Domain Containing 1	RPUSD1	3.6
234725_S_AT	Sema Domain, Immunoglobulin Domain (Ig), Transmembrane Domain (Tm) And Short Cytoplasmic Domain, (Semaphorin) 4b	SEMA4B	2.5
243768_AT	Sumo1/Sentrin Specific Peptidase 6	SENP6	2.5
209253_AT	Sorbin And SH3 Domain Containing 3	SORBS3	3.0
232493_AT	Signal Peptidase Complex Subunit 1 Homolog (S. Cerevisiae)	SPCS1	2.5
243198_AT	Testis Expressed Sequence 9	TEX9	2.8
232528_AT	Ubiquitin-Conjugating Enzyme E2e 3 (UBC4/5 Homolog, Yeast)	UBE2E4P	2.8
240155_X_AT	Zinc Finger Protein 493	ZNF493	2.5

## Discussion

The regulation of viral and cellular transcripts is of great importance, particularly with respect to latent viruses that persist in the human host for extended periods of time. In the case of adult T-cell leukemia, leukemic cells are infected with HTLV-I and contain the proviral genome, but there is extremely little expression of viral transcripts. Despite being a non-structural protein that localizes to the nucleolus, the HTLV-I accessory protein p30 is required for efficient infection in vivo [19,24]. Recent studies by our group and others have since shown that p30 acts as a negative regulator of virus expression by inhibiting the nuclear export of tax/rex mRNA [17,18]. Additional studies have reported that p30 is a transcriptional inhibitor of the viral LTR. In addition to regulating the export of tax/rex mRNA to the cytoplasm, p30 expression modifies a number of signalling pathways such as TLR4, CREB, and GSK3β [22,51,52]. How p30 accomplishes these changes in the cell is currently unknown. p30 has, however, been documented to facilitate transcription from cellular and viral promoters in conjunction with the transcriptional coactivator p300/CBP [51]. Thus, similar to another nucleolar protein, nucleolin, p30 possesses both transcriptional and posttranscriptional activities. The combination of these effects likely decrease Tax and other viral antigen expression, possibly permitting HTLV-I infected cells to remain hidden from the immune response.

In this study, we used a genome wide analysis to investigate the effect of p30 on host cell gene regulation and found a number of cellular transcripts to be increased or decreased (Tables 1, 2; see Additional files 1 and 2). These genes belonged to a variety of families, including transcriptional/translational control, cell cycle, DNA replication and repair, and cell signalling. While it is not yet known whether changes in expression of any of these genes identified here, alone or in combination, are required for cell transformation induced by HTLV-I, some have been shown to have altered expression patterns in acute ATL, such as PDCD4, 90 kDa heat-shock protein, RNA polymerase II (DNA directed), regulator of G-protein signalling, general transcription factor IIH, and Bcl-3 [34,36,37]. Considering the possibility that p30 plays a role in the onset of ATL in HTLV-I-infected individuals, some of the genes regulated by p30 may be involved in the process of cell transformation.

Using a different experimental approach which relied upon long-term stable expression of p30 in Jurkat lymphocytes, it was previously shown that p30 alters the general abundance of a number of cellular genes [23]. Several of the transcripts shown previously to be down-regulated were indeed seen in our array as also being negatively regulated by p30, but the difference was less than a 2.5-fold change in expression. The differences between these reports are not surprising given the differences in the methodology and cell lines used for these two experiments. While our study used a short-term lentiviral infection and co-transduction of peripheral blood mononuclear cells, Michael et al. used a long-term lentiviral transduction of Jurkat T-lymphocytes. It is also worthwhile to consider that long-term p30 expression has been documented to induce cell cycle alterations, which may also lead to different changes in gene expression [21]. Considering the differences, both studies are beneficial to help understand the role that p30 plays in modulating gene expression.

The main objective of this study was to evaluate whether any cellular genes were regulated post-transcriptionally by p30, in much the same way that p30 regulates Tax expression by preventing tax/rex mRNA export to the cytoplasm. While it is not yet known exactly how p30 inhibits the export of tax/rex mRNA, we hypothesized that p30 would alter the cytoplasmic abundance of cellular transcripts. In fact, we observed a number of cellular transcripts that showed either a decreased or increased abundance in the cytoplasm (Tables 3, 4). While none of these genes were previously identified as being regulated in ATL samples, this was to be expected since in our experimental approach the total abundance of the genes characterized in Tables 3 and 4 was unchanged and only cytoplasmic abundance was affected.

So how does p30 inhibit mRNA export into the cytoplasm? It is possible that p30 somehow modulates the activity of a cellular export mechanism. If this were the case, this might explain why a variety of cellular transcripts were altered in cytoplasmic abundance, indicative of a global effect. Alternatively, p30 may bind to mRNA transcripts to prevent their association with nuclear export proteins, and cellular transcripts that are inhibited may share sequence or secondary structural similarities with the tax/rex mRNA. It has been hypothesized that p30 may function by binding to both RNA and to Rex, another HTLV-I protein that is conversely responsible for up-regulating tax/rex mRNA export from the nucleus [20]. Indeed, there is evidence to suggest that p30 does bind to RNA [17,20], and that p30 might recognize a particular RNA sequence that is present in the tax-rex message, a short 150 base pair response element present specifically at the tax/rex splice junction [17]. Whether these interactions are required for the inhibition of tax/rex mRNA export is not currently known. In the current study, Rex was not expressed, suggesting that p30 can function in the absence of Rex. As a result, it is more likely that p30 might have a broad mechanism of action that applies to a number of transcripts.

On the other hand it is also possible that p30 directly or indirectly alters regulation or function of RNA export machinery. In this way p30 may actually only alter the expression or export of a few genes. The altered expression of these, in turn, might then be required for the normal expression of remaining transcripts shown to be altered in the presence of p30. Future work is required to examine the effects of p30 at the level of protein expression of candidate proteins. Another possibility explaining the function of p30 involves the binding and alteration of known cellular proteins involved in RNA modification and transport. Again, if p30 alters a known cellular mRNA trafficking pathway, it would not come as a surprise to observe so many cellular RNA transcripts as having altered expression patterns, both in total RNA samples and in cytoplasmic fractions.

Following binding to RNA, p30 might then prevent the subsequent docking and function of splicing and/or mRNA export factors. Whether p30 recognizes the sequence of this region or recognizes a secondary RNA structure is not known. Indeed, such regulation has been reported for how the retroviral HTLV Rex protein and HIV Rev protein interact with their respective RNA binding sites [53,54]. While there are no sequence similarities between the Rex responsive element (RexRE) and the Rev responsive element (RRE), HIV Rev functionally interacts with both sites while Rex is specific for the RexRE. Computer prediction of secondary structures of numerous mRNA transcripts using specific software is not an easy task but warrants future study.

Whether p30 interacts with RNA in a sequence-specific or secondary structure-specific manner, there may exist similarities between the tax/rex message and those transcripts down-regulated in the cytoplasm (Additional file 3), and examining these similarities will likely be the subject of future work. In addition, such an analysis may lead to the identification of an RNA binding motif in the p30 protein. If such a motif were found, it could also possibly lead to identification of therapeutic agents that could target such a binding motif in p30. By impairing the function of p30, one could hope to break latency and increase Tax expression in HTLV-infected patients, leading to the increased detection of infected cells by the immune system and the eventual clearance of infected cells from the body. This could perhaps provide an effective therapy for HTLV-I-infected individuals, thereby protecting patients from developing ATL later in life.

## Conclusion

In conclusion our data demonstrate that the expression of numerous cellular genes is affected by HTLV-I p30 either at the transcriptional or at the post-transcriptional (nuclear export) level suggesting that p30 may alter normal cellular homeostasis and favour transformation.

## Methods

### Lentivirus Production and Transduction

The lentiviral HIV-based vector pHR'CMV containing HTLV-I p30, the CMV-driven HIV helper virus deleted for the *env *and *nef *genes(pDNL6), and the HIV long terminal repeat-vesicular stomatitis virus G (pVSV-G) envelope were described previously [17,55]. Semi-confluent 293 T cells were transfected with packaging plasmid pDNL6, pHR'CMV:myc-p30 or pHR'CMV, and pVSV-G using calcium phosphate (Invitrogen) according to the manufacturer's instructions. At 48 hours post-transfection, cells were washed thoroughly and co-cultured with freshly isolated PBMCs. Cells were allowed to co-culture for another 48 hours, and only the non-adherent PBMCs present in the supernatant were harvested, to separate them from adherent 293 T producer cells. Myc-p30 expression was evaluated by immunofluorescence. Infected suspension cells were plated onto glass coverslips by centrifugation, fixed with 4% paraformaldehyde, permeabilized with 0.5% Triton-X-100, and stained with anti-myc (9E10; Santa Cruz Biotechnology), and anti-mouse-Alexa546 secondary (Molecular Probes). Cells were co-stained with DAPI, mounted on glass slides and imaged using a Nikon Eclipse Ti-S inverted fluorescent microscope.

To assess the rate of lentiviral infection, an assay using GFP in place of the myc-p30 gene was used (pHR'CMV:GFP). 293 T cells were transfected for 48 hours and co-cultured as described above, and cells were harvested after 48 hours. Cells were fixed in 4% paraformaldehyde and analyzed for GFP expression by flow cytometry using a FACScalibur (BD Biosciences). To determine the rate of virus production from mock producer cells or myc-p30 lentivirus producer cells, 293 T cell supernatants were harvested at 48 hours post transfection and analyzed by western blotting with antisera against HIV p24 (NIH AIDS Research and Reference Reagent Program).

### RNA Extraction and analysis

RNA from total cell extracts was isolated using Trizol (Invitrogen) according to the manufacturer's instructions. Cytoplasmic RNA was isolated by resuspending cells in a hypotonic lysis buffer (10 mM HEPES pH7.9, 1 mM MgCl2, 0.5 mM CaCl_2_, 20 mM Ribosyl complex, and 1000 U/mL of RNaseOUT (Invitrogen)), and incubated in ice for 15 min. NP-40 was then added to a final concentration of 0.27% and the lysate was immediately vortexed for 30 seconds. Cells were centrifuged, the supernatant (cytosolic) fraction was harvested, and proteinase K (50 ug/ml) was added and incubated at 37°C for 30 min. RNA was extracted twice with an equal volume of chloroform, and the final supernatant was precipitated with an equal volume of isopropanol containing 300 mM sodium acetate at -20°C. RNA samples were washed twice with 70% ethanol, and the RNA pellet was resuspended in 100 ul DEPC-H_2_O containing RNaseOUT (Invitrogen) and ribosyl vanadayl complex (Sigma). Samples were then treated with DNase (10 U/ul) for 1 hour at 37°C, and extracted with chloroform. Extracted aqueous fractions were precipitated with an equal volume of isopropanol overnight at -20°C. RNA samples were centrifuged, RNA pellets were washed twice with 70% ethanol, and resuspended in DEPC-H_2_O containing RNaseOUT (Invitrogen). RNA was quantified using a spectrophotometer and then stored at -80°C.

To assess the purity of cytoplasmic and nuclear RNA fractions, protein lysates from the cytoplasmic or nuclear fractions following cytoplasmic RNA extraction were analyzed by western blotting with anti-Cyclin A (H-432; Santa Cruz Biotechnology) or anti-Bax (N20; Santa Cruz Biotechnology).

### Microarray Analysis

RNA samples were analyzed by microarray analysis in duplicate using an Affymetrix high-density chip, HG-U133 (Plus-2). Microarray hybridization and detection were performed as described in the Affymetrix GeneChip Expression Analysis Technical Manual. Microarray data analysis was performed according to the Affymetrix GeneChip Expression Analysis (Data Analysis Fundamentals) by the K-INBRE Bioinformatics Core (Kansas IDeA Network of Biomedical Research Excellence). Minimum information about a microarray experiment (MIAME) criteria were met [56], and the complete microarray datasets described can be found at the National Center for Biotechnology Information Gene Expression Omnibus  (accession number: GSE16098). Functional annotation of transcripts was performed using the Database for Annotation, Visualization, and Integrated Discovery (DAVID) [57].

## Authors' contributions

SG and JMT performed the experiments. JMT analyzed the data and wrote the manuscript. CN developed the project, analyzed the data and wrote the manuscript. All authors have read and approved the final manuscript.

## Supplementary Material

Additional file 1 **Supplemental table 1**. Genes Downregulated >1.5 Fold in Total RNA in the presence of p30. Click here for file

Additional file 2 **Supplemental Table 2**. Genes upregulated >1.5 Fold in Total RNA in the presence of p30. Click here for file

Additional file 3 **Supplemental Table 3**. Genes Downregulated >1.5 Fold in the cytoplasm in the presence of p30. Click here for file

Additional file 4 **Supplemental Table 4**. Genes Upregulated >1.5 Fold in the cytoplasm in the presence of p30. Click here for file
